# Current status of KRAS G12C inhibitors in NSCLC and the potential for combination with anti-PD-(L)1 therapy: a systematic review

**DOI:** 10.3389/fimmu.2025.1509173

**Published:** 2025-04-15

**Authors:** Fan Zhang, Banglu Wang, Menghuan Wu, Liwen Zhang, Mei Ji

**Affiliations:** Department of Oncology, The Third Affiliated Hospital of Soochow University, Changzhou, China

**Keywords:** non-small cell lung cancer (NSCLC), targeted therapies, KRAS G12C inhibitors, anti-PD-(L)1 therapies, combination therapy

## Abstract

In recent years, precision medicine for non-small cell lung cancer (NSCLC) has made significant strides, particularly with advancements in diagnostic and therapeutic technologies. Targeted 7therapies and Anti-PD-(L)1 Therapies have emerged as vital treatment options, yet KRAS mutations, especially KRAS G12C, have been historically difficult to address. Due to the unique activation mechanism of KRAS G12C has led to the development of specific inhibitors, such as AMG 510 and MRTX849, which show promising therapeutic potential. However, results from the CodeBreaK 200 Phase III trial indicated that AMG 510 did not significantly improve overall survival compared to docetaxel. Resistance after prolonged use of KRAS G12C inhibitors continues to pose a challenge, prompting interest in new drugs and combination strategies. KRAS mutations can impair tumor-infiltrating T cell function and create an immunosuppressive tumor microenvironment, making the combination of KRAS G12C inhibitors with anti-PD-(L)1 therapies particularly appealing. Preliminary data suggest these combinations may enhance both survival and quality of life, though safety concerns remain a barrier. Ongoing research is crucial to refine treatment regimens and identify suitable patient populations. This review focuses on the development of KRAS G12C inhibitors in monotherapy and combination therapies for NSCLC, discussing major clinical trials and future research directions.

## 1 Introduction

Lung cancer is the most prevalent cancer globally and the leading cause of cancer-related mortality. In 2024, it is estimated that the United States will record 234,580 new lung cancer cases and 125,070 deaths, highlighting a substantial public health challenge (1). Non-small cell lung cancer (NSCLC) represents approximately 85% of lung cancer cases (2). Extensive research has identified several common driver mutations in NSCLC, including amplifications of the epidermal growth factor receptor (EGFR), anaplastic lymphoma kinase (ALK) fusions, mutations in the Kirsten rat sarcoma viral oncogene homolog (KRAS), BRAF V600E mutations, and ROS proto-oncogene 1 receptor tyrosine kinase (ROS1) fusions. Although KRAS mutations are less prevalent than EGFR alterations, they are present in 25-30% of NSCLC cases (3). The prevalence of KRAS mutations in NSCLC varies based on factors such as geographic location and smoking history. For instance, the mutation rate in U.S. lung adenocarcinoma patients is significantly higher than in Asian patients (approximately 35% vs. 13%) (4). Smoking status is also a key determinant, with KRAS mutations being most common among former or heavy smokers (4, 5). Regarding gender, while some studies suggest a slightly higher overall mutation rate in women, the gender distribution in specific subtypes such as KRAS G12C appears to vary across racial groups, warranting further confirmation (6, 7). The RAS family functions as signal transducers, with conserved N-terminal and highly variable C-terminal domains. As a key member of this family, KRAS is integral to signal transduction pathways (8). KRAS mutations in cancer predominantly occur in codons 12, 13, or 61 (9). In NSCLC, mutations in the glycine residue at codon 12 (G12) represent over 80% of KRAS mutations, followed by mutations at G13 and Q61. The KRAS G12C mutation is the most prevalent, accounting for approximately 39% of all KRAS mutations in NSCLC (10, 11).

KRAS has long been considered an undruggable target (12), and most patients with advanced KRAS-mutant NSCLC currently receive conventional platinum-based chemotherapy, which is associated with poor prognosis. In recent years, significant progress has been made in developing inhibitors targeting KRAS G12C, with AMG 510 (sotorasib) and MRTX849 (adagrasib) advancing most rapidly. These two inhibitors were approved by the U.S. Food and Drug Administration (FDA) on May 18, 2021, and December 13, 2022, respectively. However, AMG 510 faced challenges in phase III trials. The CodeBreaK 200 trial (NCT04303780) demonstrated that the median progression-free survival (PFS) was 5.6 months for 169 patients treated with AMG 510, compared to 4.5 months for 174 patients treated with docetaxel; despite this, there was no significant difference in median overall survival (OS) between the groups, drawing some criticism from the FDA (13). New KRAS G12C inhibitors such as D-1553 (Garsorasib), IBI351 (Fulzerasib), and JAB-21822 (Glecirasib) have shown promising results in clinical trials, with further research ongoing (14–16). The efficacy of KRAS G12C inhibitors has encountered limitations, with challenges related to drug resistance persisting (17). To address resistance, various combination therapies with KRAS G12C inhibitors are being tested, including combinations with immune checkpoint inhibitors, chemotherapeutic agents, MEK inhibitors, and SHP2 inhibitors.

Programmed death receptor 1 (PD-1), a member of the immunoglobulin superfamily first identified by Honjo’s team in 1992, is primarily expressed on the surface of activated T cells (18). When PD-1 binds to its ligand, PD-L1, it drives immune evasion by suppressing T-cell receptor (TCR) signaling, reducing the secretion of cytotoxic molecules such as IFN-γ, and inducing T-cell exhaustion (19, 20). PD-1/PD-L1 inhibitors, such as Pembrolizumab and Nivolumab, restore T-cell activity by blocking the PD-1/PD-L1 interaction, thereby reactivating the anti-tumor functions of T cells. These agents have become cornerstone therapies for solid tumors, including NSCLC (19, 20). Given the significant immunomodulatory properties of KRAS G12C inhibitors, which can reshape the immune system and bolster anti-tumor responses, anti-PD-(L)1 therapies may achieve a synergistic effect when combined with KRAS G12C inhibitors. Currently, multiple KRAS G12C inhibitors are exploring combination use with anti-PD-(L)1 therapies to address the many challenges these drugs face (21, 22). By summarizing existing literature, conference reports, and clinical trial data, our study discusses the epidemiology of KRAS G12C Mutant NSCLC, the oncogenic mechanisms of KRAS mutations, and their immune impact. This study focuses on the efficacy and safety of KRAS G12C inhibitors both as monotherapies and in combination therapies, including anti-PD-(L)1 treatments, incorporating the latest clinical research findings to provide new insights and perspectives for clinical and research professionals.

## 2 Epidemiology of and biology of KRAS G12C in NSCLC

Statistics indicate that KRAS mutations in cancer predominantly occur at codons 12, 13, or 61 (9). In NSCLC, mutations at codon 12 account for over 80%, followed by mutations at codons 13 and 61. Among these, the codon 12 mutations include KRAS G12C, G12D, and G12V, with KRAS G12C being the most common. It represents approximately 39% of all KRAS mutations in NSCLC (10, 11). Additionally, KRAS G12C in NSCLC is more frequently observed in Caucasian and African American patients compared to Asian patients. Notably, in Caucasian populations, the frequency of KRAS G12C is higher in females than in males, while the reverse is true among Asian patients (6). Furthermore, KRAS G12C is typically more prevalent in patients with a history of smoking (23).

**Figure 1:**
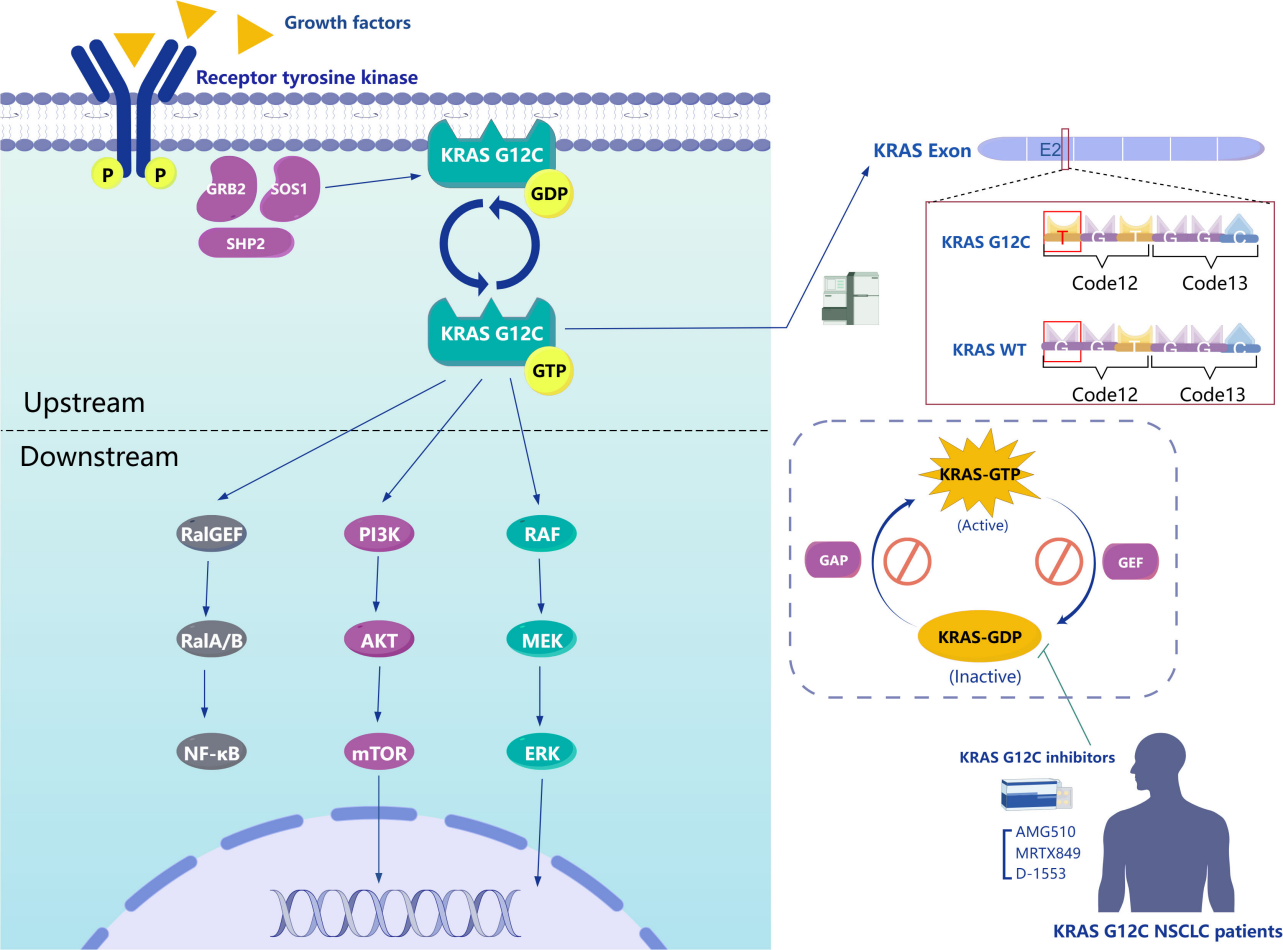
Left Panel, The intracellular signaling pathways of KRAS G12C. Upper Right Panel, Mechanism of KRAS G12C mutation. Lower Right Panel, The interconversion between KRAS-GTP and KRAS-GDP bound states and mechanism of action for representative inhibitors of KRAS G12C.

The rat sarcoma virus oncogene (RAS) family functions as a conserved GTPase signal transducer, with a conserved N-terminal domain and a highly variable C-terminal domain, playing a crucial role in signal transduction (8). The oncogene KRAS, which is pathogenic in various cancers, is a member of the RAS gene family. There are two types of KRAS genes, KRAS1 and KRAS2, located on the short arm of chromosome 6 and chromosome 12, respectively. The KRAS protein, with 188 amino acids and a molecular weight of 21.6 kDa, comprises a catalytic domain known as the G-domain and a hypervariable region (HVR). The G-domain includes a phosphate (P) loop, along with two switches, Switch I and Switch II, while the HVR contains the CAAX motif related to membrane localization (24, 25). KRAS acts as a “molecular switch” in signal transduction due to its GTPase activity, toggling between an inactive state when bound to GDP and an active state when bound to GTP. This activity regulates signal transduction from activated membrane receptors to intracellular molecules. Upon stimulation by extracellular signals such as growth factors (e.g., epidermal growth factor receptor, insulin-like growth factors), hormones, cytokines, and neurotransmitters, upstream molecules are activated. GTPase-activating proteins (GAPs) and guanine nucleotide exchange factors (GEFs) regulate this process by mediating the conversion between KRAS-GTP and KRAS-GDP states, thereby activating downstream effectors and signal transduction pathways associated with cell proliferation and survival (**Figure 1**) (25, 26). Conversely, when KRAS is mutated, GAPs cannot effectively enhance the GTPase catalytic rate, reducing its ability to hydrolyze GTP. This mutation keeps KRAS in an active GTP-bound state, leading to continuous activation of downstream signaling pathways, as KRAS maintains a high affinity for GTP. Furthermore, the structural feature of wild-type KRAS, lacking a sufficiently large surface pocket, prevents potential small molecule inhibitors from binding, historically rendering KRAS undruggable (27). However, KRAS G12C, where the 12th amino acid glycine is replaced by cysteine, represents a breakthrough (**Figure 1**). Unlike other mutations, KRAS G12C, located on the P-loop, exhibits GTPase activity similar to wild-type KRAS (28). Nevertheless, KRAS G12C leads to sustained downstream activation due to its stabilized nucleotide state, which reduces the nucleotide cycling rate and promotes continuous cell proliferation rather than affecting the GDP-GTP exchange rate (29). This characteristic positions KRAS G12C research as a breakthrough in targeting the historically challenging KRAS target. Eventually, a team discovered a small molecule capable of irreversibly binding to KRAS G12C by targeting the cysteine formed due to the mutation. This small molecule creates a new pocket beneath the Switch II region, keeping KRAS in a GDP-bound state, which impedes its effective binding with downstream effectors like RAF (**Figure 1**) (30). This research has ushered in a new era in targeting KRAS.

## 3 Advancements and clinical impact of KRAS G12C inhibitors

Subsequently, extensive research has continually enhanced the inhibitory effects on KRAS G12C, leading to the development of a series of KRAS G12C inhibitors such as ARS-853, ARS-1620, AMG 510, and MRTX859, among others. ARS-853 and ARS-1620, among the first KRAS G12C inhibitors developed, have not yet entered clinical trials. Most KRAS G12C inhibitors have begun clinical trials, with two main drugs taking center stage: AMG 510 (31) and MRTX849 (32). Additionally, clinical trials for several novel KRAS G12C inhibitors have yielded varying degrees of results. The efficacy and safety data from these trials are summarized in **Table 1**. Furthermore, various KRAS G12C inhibitors are being tested in combination with non-traditional therapies, such as chemotherapy and immunotherapy, with the specific combination regimens detailed in **Table 2**.

**Table 1:** Clinical trials of novel KRAS G12C inhibitor in NSCLC.

KRAS G12C inhibitors	Trial/Trial number	Phase	No. of NSCLC pts	Interventions	Results	Primary Adverse Reactions and incidence of Grade 3 or Higher Adverse Events
D-1553 (Garsorasib)	NCT05383898	I	79	600mg BID (62/79)	ORR 38.7% DCR 90.3%	Elevated liver enzymes, nausea, vomiting 49.6%
		II	123	600mg BID	ORR 49.6% (95% CI, 40.5-58.8) DCR 88.6% (95% CI, 81.6-93.6) mPFS 7.56mo (95% CI, 5.55-9.69) mDOR 12.78mo (95% CI, 6.21-NE)	
	NCT05379946	II	33	D-1553 +Ifebemtinib	ORR 87.5% DCR 93.8%	Diarrhea, enteritis, peripheral edema, proteinuria
IBI351 (GFH925)	NCT05005234	I	74	600mg BID (31/74)	ORR 50.0% (95% CI, 31.3-68.7) DCR 96.7% (95% CI, 82.8-99.9) mPFS 5.5mo (95% CI, 5.3-6.8)	Anemia, elevated liver enzymes, fatigue, proteinuria 41.4%
		II	116	600mg BID	ORR 49.1% (95% CI, 39.7-58.6) DCR 90.5% (95% CI, 83.7-95.2) mPFS 9.7mo (95% CI, 5.6-11.0)	
	NCT05756153	II	20	IBI351 +cetuximab	ORR 80.0% DCR 100%	
JAB-21822 (Glecirasib)	NCT05009329	I/II	22	400-800mg QD	ORR 70% (7/10) DCR 100% (10/10)	Anemia, elevated serum bilirubin, elevated liver enzymes, hypertriglyceridemia 39.5%
		II	119	800mg QD	ORR 47.9% (95% CI, 38.5%-57.3%) DCR 86.3% (95% CI, 78.7%-92%) mPFS 8.2mo (95% CI, 5.5-13.1) mOS 13.6mo (95% CI, 10.9-NE) mDOR (95% CI, 7.2 – NE)	
	NCT05288205	I/IIa	102	JAB-21822 +JAB-3312	ORR 64.7% DCR 93.1% mPFS 12.2mo	Anemia, elevated liver enzymes, hypertriglyceridemia, elevated bilirubin 43.8%
JDQ443 (Opnurasib)	KontRASt-01 (NCT04699188)	Ib/II	38	200mg BID (11/38)	ORR 54.5%	Fatigue, edema, diarrhea, nausea, vomiting, peripheral neuropathy 7.1%
		Ib/II	24	JDQ443 +TNO155	ORR 33.3% DCR 66.7%	36%
LY3537982 (Olomorasib)	LOXO-RAS-2000 (NCT04956640)	I/II	58	50-200mg BID	ORR 39% DCR 73% mPFS 6.0mo (95% CI, 3-NE)	Diarrhea, fatigue, nausea 5%
GDC-6036 (divarasib)	NCT04449874	I	60	50-400mg QD	ORR 53.4% (95% CI, 39.9-66.7) mPFS 13.1mo (95% CI, 8.8-NE)	Nausea, diarrhea, vomiting, fatigue, loss of appetite, elevated liver enzymes 12%
HS-10370	HS-10370-101 (NCT05367778)	I	43	400-1600mg QD (41/43)	ORR 51.2% (21/41) DCR 95.1% (39/41) mPFS 11.3mo (95% CI, 6.1-NA)	Elevated liver enzymes, anemia, diarrhea, weight gain, decreased appetite, hypoproteinemia, nausea, fatigue, rash 27.3%
BI-1823911	NCT04973163	I		50-1200mg QD		Diarrhea, nausea, vomiting 47.1%(8/17)
MK-1084	NCT05067283	I				
JNJ-74699157	NCT04006301	I	5	100-200mg QD	Terminated early due to toxicity	Elevated serum creatine phosphokinase 60%

ORR (Objective Response Rate), The proportion of patients with a partial or complete response to therapy; DCR (Disease Control Rate), The percentage of patients who have achieved complete response, partial response, and stable disease; mPFS (Median Progression-Free Survival), The median length of time during and after treatment that a patient lives with the disease without it getting worse; mDOR (Median Duration of Response), The median length of time that a tumor continues to respond to treatment without growth.

**Table 2:** Clinical trials of KRAS G12C inhibitors combined with pathway-targeted agents in NSCLC.

KRAS G12C inhibitors	Mechanism of Action	Drugs	Trial number	Phase
AMG 510	Chemotherapy	Cisplatin/Carboplatin + Pemetrexed	NCT05118854	II
		Carboplatin + Pemetrexed	NCT04185883	Ib
	Anti-EGFR antibody	Panitumumab	NCT05993455	II
		Tarloxotinib	NCT05313009	Ib/II
	EGFR-TKI	Afatinib	NCT04185883	Ib
	SHP2 inhibitor	RMC-4630	NCT05054725	II
		JAB-3312	NCT04720976	I/IIa
		ERAS-601	NCT04959981	Ib
		BBP-398	NCT05480865	I
	RAF/MEK inhibitor	Avutometinib	NCT05074810	I/II
	MEK inhibitor	Trametinib	NCT04185883	Ib
	ERK inhibitor	ASN-007	NCT04959981	Ib
	mTOR inhibitor	Everolimus	NCT04185883	Ib
MRTX849	Chemotherapy	Cisplatin/Carboplatin + Pemetrexed	NCT05609578	II
	Anti-EGFR antibody	Cetuximab	NCT06024174	I/II
	EGFR-TKI	Afatinib	NCT03785249	I/II
	SHP2 inhibitor	TNO155	NCT04330664	I/II
		RMC-4630	NCT04418661	I/II
		BMS-986466	NCT06024174	I/II
	SOS1 inhibitor	MRTX0902	NCT05578092	I/II
	RAF/MEK inhibitor	Avutometinib	NCT05375994	I/II
	mTOR inhibitor	Sirolimus	NCT05840510	I/II
	CDK4/6 inhibitor	Palbociclib	NCT05178888	I/Ib
	FAK inhibitors	KO-2806	NCT06026410	I
D-1553	SHP2 inhibitor	GH21	NCT06435455	Ib/II
	FAK inhibitors	Ifebemtinib	NCT05379946	Ib/II
			NCT06166836	I/II
IBI351	Anti-EGFR antibody	Cetuximab	NCT05756153	II
JAB-21822	SHP2 inhibitor	JAB-3312	NCT05288205	I/IIa
			NCT06416410	III
JDQ443	Anti-EGFR antibody	Cetuximab	NCT05358249	Ib/II
	SHP2 inhibitor	TNO155	NCT04699188	Ib/II
	MEK inhibitor	Trametinib	NCT05358249	Ib/II
	CDK4/6 inhibitor	Ribociclib	NCT05358249	Ib/II
LY3537982	Chemotherapy	Cisplatin/Carboplatin + Pemetrexed	NCT04956640	I/II
GDC-6036	Anti-EGFR antibody	Cetuximab	NCT04449874	Ia/Ib
	EGFR-TKI	Afatinib	NCT04449874	Ia/Ib
	SHP2 inhibitor	Migoprotafib	NCT04449874	Ia/Ib
	PI3Kα inhibitor	Inavolisib	NCT04449874	Ia/Ib
BI-1823911	Pan-KRAS inhibitor	BI-1701963	NCT04973163	Ia/Ib

For each combination therapy regimen, the target, therapeutic agents, trial identifier, and trial phase are specified.

### 3.1 Early-stage investigational drugs

**Figure 2:**
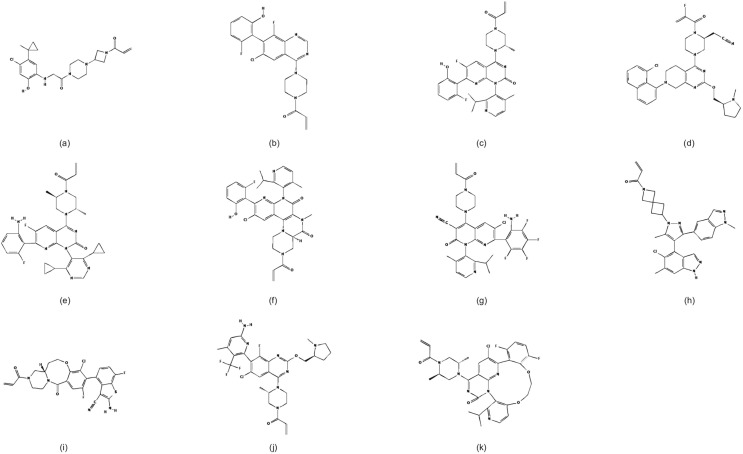
Chemical structures of KRAS G12C inhibitors. **(a)** ARS-853, **(b)** ARS-1620, **(c)** AMG 510, **(d)** MRTX849, **(e)** D-1553, **(f)** IBI351, **(g)** JAB-21822, **(h)** JDQ443, **(i)** LY3537982, **(j)** GDC-6036, **(k)** MK-1084. Adapted from PubChem (https://pubchem.ncbi.nlm.nih.gov/).

The development of KRAS G12C inhibitors began with preclinical studies. ARS-853 was the first inhibitor identified to selectively inhibit intracellular KRAS, with studies demonstrating its low micromolar potency in cells and its ability to efficiently target KRAS G12C bound to GDP (**Figure 2**) (33). However, subsequent research revealed that ARS-853 is inactive *in vivo*, and it is currently used only as a tool compound. ARS-1620, a newly developed KRAS G12C inhibitor, shares the same mechanism of action as ARS-853 but exhibits better bioavailability (**Figure 2**). Unlike ARS-853, ARS-1620 specifically targets the KRAS G12C mutation both *in vivo* and ex vivo (34). However, ARS-1620 has not advanced to clinical trials. Although ARS-1620 was reported to inhibit KRAS G12C and block KRAS-GTP formation ex vivo, residual KRAS activity was observed in live cells (35). Some studies have found that ARS-1620 effectively inhibits tumor growth both *in vivo* and ex vivo when used in combination with mTOR, IGF1R, and SHP2 inhibitors (36, 37). Plans for these combination regimens were put on hold due to the limitations of ARS-1620, which never progressed to clinical trials. Despite these preclinical inhibitors not fully addressing the clinical challenges, they provided valuable insights and evidence for the development of future drugs.

### 3.2 Frontline drugs

#### 3.2.1 AMG 510 (Sotorasib)

AMG 510, developed by Amgen, is the first FDA-approved inhibitor of KRAS G12C. It shares a similar ligand structure with ARS-1620, specifically binding to the Switch II pocket of KRAS G12C that binds GDP, using the acrylamide portion for slow binding to cysteine-12 (38). Compared to ARS-1620, AMG 510 has a highly optimized methylisopropylpyridine substituent, enhancing its interaction by binding to the His95 groove of KRAS G12C (**Figure 2**). This optimization results in approximately 10 times higher potency than ARS-1620 (31). AMG 510 selectively inhibits KRAS G12C mutants while showing insensitivity to other subtypes or wild-type KRAS (31, 39). Additionally, AMG 510 exhibits reasonable oral bioavailability across species (38). Encouraged by these preclinical results, AMG 510 advanced to clinical trials. In the CodeBreak100 (NCT03600883) Phase I/II trial, AMG 510 demonstrated favorable efficacy and safety (40, 41), leading to its FDA approval on May 28, 2021, as the first direct inhibitor of KRAS G12C. A long-term analysis of the trial updated with the latest data included 174 previously treated patients receiving AMG 510 at a dose of 960 mg QD. Results showed an objective response rate (ORR) of 41%, a disease control rate (DCR) of 84%, a median PFS of 6.3 months, an OS of 12.5 months, a median duration of response (DOR) of 12.3 months, a 1-year OS of 51%, and a 2-year OS of 33%, all significantly higher than previous results with pemetrexed and docetaxel (42, 43). However, 70% of patients experienced treatment-related adverse events (TRAEs), with 21% being grade 3 or higher. The most common TRAEs were diarrhea (30%), elevated alanine aminotransferase (ALT) levels (18%), and elevated aspartate aminotransferase (AST) levels (18%) (43). The results of the Phase III AMG 510 vs. docetaxel trial were less encouraging. The CodeBreaK 200 (NCT04303780), a randomized, controlled Phase III trial, compared AMG 510’s efficacy and safety to docetaxel in previously treated patients with advanced KRAS G12C-mutated NSCLC. Median PFS for the AMG 510 group improved to 5.6 months over the 4.5 months of the docetaxel group, but OS did not improve (13). Despite better quality of life and reduced toxicity, survival outcomes were underwhelming, leading to a critical analysis by the FDA’s Oncologic Drugs Advisory Committee, casting uncertainty on AMG 510’s clinical value and necessitating further evidence for continued market presence.

AMG 510 is being explored in various combination therapies, demonstrating promising efficacy and manageable safety. In the CodeBreaK 101 trial, AMG 510 combined with platinum-doublet chemotherapy showed notable results in NSCLC patients, with a 65% ORR, a median PFS of 10.8 months in first-line therapy, and a 42% ORR in second-line and beyond treatment (44). Combinations with other agents are also under investigation, including MEK inhibitors (trametinib) and pan-ErbB inhibitors (afatinib), achieving ORRs of 20%-34.8% and DCRs up to 87% (45, 46). Ongoing trials are evaluating AMG 510 with Raf/MEK and SHP2 inhibitors, aiming to expand its therapeutic potential (47).

#### 3.2.1 MRTX849 (Adagrasib)

MRTX849 is another well-studied irreversible inhibitor with a mechanism of action similar to that of AMG 510 (**Figure 2**) (32, 48). It has been shown that MRTX849 exhibits higher activity among a wide range of FDA-approved covalent drugs and offers greater stability compared to AMG 510 (49). Clinical results from the KRYSTAL-1 Phase I/II study, which evaluates the efficacy and safety of MRTX849, demonstrated favorable outcomes (50). Two-year follow-up data from the KRYSTAL-1 study reinforce MRTX849’s role in previously treated patients with KRAS G12C-mutated NSCLC. As of January 1, 2023, 132 patients treated with MRTX849 had a median OS of 14.1 months and a median PFS of 6.9 months. About one-third of patients achieved durable two-year efficacy, with a safety profile consistent with previous reports, and no new safety signals identified (51). This long-term study further supports MRTX849 as a promising candidate for further investigation. The KRYSTAL-12 Phase III study (NCT04685135) is currently recruiting patients to compare the efficacy of MRTX849 (600 mg BID) with docetaxel (52). This study aims to determine if MRTX849 yields different results from AMG 510, potentially advancing the development of KRAS G12C inhibitors. Notably, MRTX849 has demonstrated the ability to combat brain metastases, which may not be the case with AMG 510. NSCLC patients with KRAS G12C are more prone to developing brain metastases (53). Preclinical brain metastasis modeling studies have shown that MRTX849 achieves high cerebrospinal fluid concentrations, with an unbound brain to unbound plasma concentration ratio of 1 at a 200 mg/kg dose level after 8 hours, indicating strong blood-brain barrier penetration (53). This study highlights MRTX849’s therapeutic potential in the central nervous system and its implications for developing additional KRAS G12C inhibitors targeting brain metastases. The KRYSTAL-1 trial also included cohorts evaluating MRTX849 in patients with CNS metastases from NSCLC with KRAS G12C mutations. Imaging indicated an ORR of 42%, a DCR of 90%, a median PFS of 5.4 months, and a median OS of 11.4 months among MRTX849-treated patients. The treatment regimen was consistent with other cohorts, with CNS-specific TRAEs including dysgeusia (24%) and dizziness (20%) (54). MRTX849 is the first KRAS G12C inhibitor prospectively demonstrated to have intracranial activity, offering efficacy against brain metastases. This positions MRTX849 to potentially surpass AMG 510 and become the first KRAS G12C inhibitor marketed in multiple regions.

Ongoing studies are enhancing MRTX849’s antitumor activity through combination therapies. In KRAS G12C-mutated colorectal cancer, combining MRTX849 with the anti-EGFR antibody cetuximab achieved an ORR of 34%, a DCR of 85%, and a median PFS of 6.9 months, with favorable outcomes and a tolerable safety profile (55). This regimen has been FDA approved for advanced colorectal cancer patients with KRAS G12C mutations. Other combinations, including SOS1 inhibitors, mTOR inhibitors, and SHP2 inhibitors, are under investigation to further expand its clinical potential (56).

### 3.3 Novel drugs

#### 3.3.1 D-1553 (Garsorasib)

D-1553 is a selective and potent oral KRAS G12C inhibitor developed by InvestisBio (**Figure 2**). It specifically binds to GDP-bound KRAS G12C proteins and has been shown to be inactive against KRAS wild-type (WT) and KRAS G12D cell lines (57). Strong specific inhibition of KRAS G12C was demonstrated in both a cellular model and a nude mouse xenograft tumor model (58, 59). Recent studies have found that D-1553 not only binds to GDP-bound KRAS G12C but also inhibits the phosphorylation of ERK and AKT signaling pathways, effectively blocking downstream signaling in KRAS G12C mutant cells. In multiple cell line experiments, D-1553 demonstrated higher potency than AMG 510 and MRTX849. The potential for D-1553 to cause tumor regression was reaffirmed in xenograft tumor model trials. It may also have a role similar to MRTX849 in targeting brain metastases (57). These findings suggest that D-1553 could be a promising KRAS G12C inhibitor. Clinical trials targeting D-1553 are also ongoing and have shown promising anti-tumor activity in patients with NSCLC with KRAS G12C mutations in phase I studies (60). Results from the phase II study were recently published. This study enrolled patients with advanced NSCLC with KRAS G12C mutations who had received prior anti-PD-(L)1 therapy and platinum-based chemotherapy, administering D-1553 at a dose of 600 mg QD. Among 123 treated patients, the ORR was 50%, the DCR was 88.6%, the median DOR was 12.78 months, and the median PFS was 7.56 months, with the median OS not yet reached. TRAEs were reported in 95% of patients, with grade 3 or higher events in 50%. No TRAEs led to the discontinuation of D-1553, and most adverse events were well-managed (14, 61). D-1553 has demonstrated high tumor response rates and long-term remission durations. With a Phase III trial comparing it to docetaxel currently recruiting patients, D-1553’s favorable efficacy is expected to continue.

In addition to monotherapy, D-1553 has also demonstrated therapeutic potential in combination with other agents. Focal adhesion kinase (FAK) inhibitors show promise in combination with KRAS inhibitors. A Phase II study combining D-1553 with the FAK inhibitor Ifebemtinib in treatment-naive NSCLC patients with KRAS G12C mutations reported an ORR of 87.5% and a DCR of 93.8%, with no additional toxicity compared to monotherapy (62). These results highlight excellent efficacy and safety, offering potential for further KRAS-G12C inhibitor development.

#### 3.3.2 IBI351 (GFH925)

IBI351 is a novel KRAS G12C inhibitor developed by Innovent Biologics (**Figure 2**). In phase I trials involving patients with advanced solid tumors, IBI351 demonstrated promising efficacy and tolerability (63). The latest update on monotherapy in metastatic colorectal cancer(CRC) indicates hopeful and durable efficacy with manageable safety (64). Preliminary results from the phase II trial for patients with advanced NSCLC have also been promising (15, 65). As of December 13, 2023, among the 116 advanced NSCLC patients participating in the treatment trial, the ORR was 49.1%, and the DCR was 90.5%. The median DOR has not been reached, and the median PFS was 9.7 months. TRAEs occurred in 92.2% of the patients, with 41.4% experiencing TRAEs of grade ≥3 (15). During the analysis of the tumor mutation profiles of treated patients, researchers identified TP53, STK11, and KEAP1 as the most common co-mutated genes with KRAS G12C. Co-mutations involving genes like STK11 and KEAP1 were associated with poorer PFS (15). It is hypothesized that selectively using KRAS G12C inhibitors based on patient gene co-mutation status might enhance the efficacy of these inhibitors, and further studies are anticipated to validate this hypothesis.

IBI351 is gaining attention for its potential in combination therapies beyond monotherapy. The KROCUS Phase II study evaluated IBI351 with the anti-EGFR antibody cetuximab in KRAS G12C-mutated patients, reporting an ORR of 80% and a DCR of 100% among 20 evaluable patients, including an ORR of 71.4% in those with brain metastases. The combination demonstrated manageable safety, with 18.5% of patients experiencing grade 3 TRAEs and no grade 4 or 5 events (66). While the results highlight strong efficacy and safety, larger studies are needed to confirm these findings.

#### 3.3.3 JAB-21822 (Glecirasib)

In early preclinical studies, JAB-21822 exhibited strong inhibitory effects on cell growth as a monotherapy across various human cancer cell lines (**Figure 2**) (67). In phase I/II studies targeting patients with advanced solid tumors harboring KRAS G12C mutations, JAB-21822 demonstrated good tolerability and significant preliminary efficacy (68). A recent phase II study in China involved 119 patients with advanced NSCLC with KRAS G12C mutations, who received 800 mg of JAB-21822 daily as monotherapy. The study showed an ORR of 47.9%, a DCR of 86.3%, a median PFS of 8.2 months, and a median OS of 13.6 months. The median DOR has not yet been reached. TRAEs of any grade were observed in 97.5% of patients, with the most common being anemia and increased blood bilirubin levels (16). Despite the relatively high incidence of TRAEs, JAB-21822 exhibited very low gastrointestinal toxicity, which is an advantage over other KRAS G12C inhibitors and may enhance patient adherence to oral therapy.

JAB-21822 combined with the SHP2 inhibitor JAB-3312 has shown synergistic effects in KRAS G12C inhibitor-resistant tumors (67). In NSCLC patients, first-line combination therapy achieved an ORR of 64.7% and a DCR of 93.1%, with a median PFS of 12.2 months in 102 patients (69). Efficacy was consistent across different PD-L1 expression levels, with ORRs ranging from 46.2% to 82.4% and median PFS values from 8.1 to 15 months. Grade ≥3 TRAEs occurred in 43.8% of patients, including anemia, elevated liver enzymes, and hypertriglyceridemia (69). A Phase III trial is ongoing to further evaluate this promising regimen.

#### 3.3.4 JDQ443

JDQ443 is a novel KRAS G12C inhibitor featuring a unique 5-methylpyrazole core and a spiro-azolide linker (**Figure 2**). This inhibitor forms novel interactions with specific residues in the SW-II pocket of KRAS G12C, bypassing the H95 residue, thereby durably and irreversibly immobilizing KRAS G12C in a GDP-bound inactive state. This represents a different binding mode compared to inhibitors such as AMG 510 and MRTX849 (70). *In vivo* studies have shown that JDQ443 achieves superior target occupancy, demonstrating favorable oral bioavailability and dose-dependent antitumor activity, as well as tolerability across various *in vitro* and *in vivo* models (71, 72). Moreover, studies suggest that JDQ443 may reverse resistance to other KRAS G12C inhibitors like MRTX849 (72). Relevant clinical studies of JDQ443 are underway. KontRASt-01 (NCT04699188) is a Phase Ib/II, multicenter clinical study evaluating the safety, tolerability, and efficacy of JDQ443. Recent data indicate that 84 patients received JDQ443 monotherapy, including 38 cases of NSCLC. At the recommended Phase I dose of 200 mg BID, 71.4% of patients experienced TRAEs of varying grades, with 7.1% experiencing Grade 3 TRAEs, and no Grade 4-5 TRAEs observed. The ORR for NSCLC patients at the recommended dose was 54.5% (73). The KontRASt-06 (NCT05445843) Phase II study is evaluating JDQ443 monotherapy as a first-line treatment for patients with advanced NSCLC with KRAS G12C mutations. The study includes two groups: one with tumor PD-L1 expression of less than 1% regardless of STK11 mutation status, and the other with PD-L1 expression of greater than or equal to 1% with STK11 co-mutation. The study is currently enrolling participants (74). The KontRASt-02 Phase III study aims to compare the efficacy of JDQ443 monotherapy against docetaxel in patients with advanced NSCLC with KRAS G12C mutations and is also recruiting participants (75).

JDQ443 combined with the SHP2 inhibitor TNO155 has shown potential to enhance therapeutic outcomes (71). In the KontRASt-01 study, among 24 NSCLC patients previously treated with a KRAS G12C inhibitor, the combination achieved an ORR of 33.3% and a DCR of 66.7%. TRAEs occurred in 88% of patients, with grade 3-4 TRAEs in 36% (76). Preclinical studies are also exploring combinations with SHP2 and MEK inhibitors, highlighting the potential of JDQ443 in combination therapies to improve efficacy (77).

#### 3.3.5 LY3537982 (Olomorasib)

LY3537982 is a novel, orally available KRAS G12C inhibitor characterized by unique pharmacological properties that achieve high target occupancy at low concentrations, offering higher inhibition efficiency of KRAS G12C compared to AMG 510 and MRTX849 (**Figure 2**). In the KRAS G12C-mutated H358 lung cancer cell line, LY3537982 exhibited significantly lower IC50 values for the inhibition of GTP-bound KRAS and phosphorylated ERK than the other two KRAS G12C inhibitors, underscoring its potent tumor growth inhibition effect (78). The LOXO-RAS-20001 study (NCT04956640), a Phase I trial investigating LY3537982 in patients with advanced solid tumors harboring KRAS G12C mutations, reported a favorable safety profile across various solid tumors, including NSCLC, CRC, and PANC. Patients receiving multiple dose gradients (50-200 mg BID) experienced no serious adverse events or deaths related to treatment (79). Recent data from a Phase I study of LY3537982 in patients with gastrointestinal tumors highlighted its preliminary efficacy and favorable safety both as a monotherapy and in combination with cyclosporine (80). These findings suggest LY3537982’s potential in both standalone and combination chemotherapeutic regimens. Updated results from the LOXO-RAS-20001 study as of October 30, 2023, included 157 patients (58 NSCLC, 32 CRC, 24 PANC, 43 other solid tumors) receiving LY3537982 (50-200 mg BID PO). Notably, among 29 NSCLC patients previously treated with KRAS G12C inhibitors, the ORR was 39%, with a DCR of 73% and a median PFS of 6 months. TRAEs occurred in 62% of patients, with the most common being diarrhea (24%), fatigue (10%), and nausea (10%) (81). These results affirm the preliminary efficacy and safety of LY3537982 and help fill the clinical trial void for this drug in NSCLC patients. The Phase II trial is currently recruiting, and the results are promising. For advanced NSCLC, combination therapies involving LY3499446 with EGFR-TKIs and CDK4/6 inhibitors have also entered clinical trial phases, showing potential for enhanced therapeutic strategies.

#### 3.3.6 GDC-6036 (divarasib)

GDC-6036 is a highly effective and selective inhibitor targeting the KRAS G12C mutation. It features a highly reactive acrylamide warhead and two densely functionalized heterocycles: quinoline and pyridine. Its structure includes a bulky, rotationally restricted C−C bond (**Figure 2**) (82). Previous studies have demonstrated that GDC-6036 exhibits dose-dependent tumor inhibition in xenograft models using pancreatic cancer cell lines. Further research has revealed that GDC-6036 can inhibit KRAS G12C alkylation and MAPK signaling pathway activity in a dose-dependent manner (83). GDC-6036 has shown promising clinical activity and high target occupancy in solid tumors with KRAS G12C mutations, with a safety profile within controllable limits (84). The latest Phase I study data indicate that among 60 NSCLC patients receiving GDC-6036 treatment at oral doses ranging from 50 to 400 mg, the ORR was 53.4%. A confirmed therapeutic response was observed in 53.4% of patients, with a median PFS of 13.1 months. Responses were also observed in other solid tumor patients within this cohort. Treatment-related adverse events occurred in 93% of patients, with no dose-limiting toxicities or treatment-related deaths reported (85). Additionally, a decrease in the KRAS G12C variant allele frequency, as assessed through sequencing of circulating tumor DNA, was associated with response (85). In colorectal cancer, combining GDC-6036 with cetuximab demonstrated antitumor activity, while studies are ongoing to explore combinations with chemotherapy, bevacizumab, GDC-1971 (SHP2 inhibitor), and inavolisib (PI3Kα inhibitor) (86). Notably, in KRAS G12C-mutant NSCLC models, GDC-6036 combined with GDC-1971 reduced tumor growth more effectively than either drug alone and was well-tolerated (87). The efficacy of combining GDC-6036 with additional drugs in NSCLC warrants further investigation.

Several novel KRAS G12C inhibitors are currently under investigation. BI-1823911, a novel inhibitor, has demonstrated efficacy comparable to 100 mg/kg doses of AMG 510 and MRTX849 in NSCLC and CRC mouse models when administered orally at 60 mg/kg daily (88). In an ongoing Phase I trial, patients with advanced solid tumors are receiving ascending doses of BI-1823911 monotherapy. Preliminary results show clinical activity, with disease control observed in 11 out of 17 patients during the early dose-escalation phase (≤100 mg), and an acceptable safety profile (89). In combination studies, the SOS1 inhibitor BI-1701963 paired with BI-1823911 has shown synergistic antitumor effects in preclinical NSCLC models, and related clinical trials are ongoing (88, 90). The 2024 AACR conference presented results from the Phase I study HS-10370-101 of the KRAS G12C inhibitor HS-10370. The study included 55 patients with advanced solid tumors, of which 43 were NSCLC patients. Across all solid tumor patients, the ORR was 49.0%, and the DCR was 89.8%. In NSCLC patients receiving daily doses of ≥400 mg, the ORR was 51.2% and the DCR was 95.1%, with the median PFS at 11.3 months and a 6-month PFS rate of 75%. Among the 55 patients, 87.3% experienced TRAEs, and 27.3% experienced grade 3 or higher TRAEs, with common adverse events including elevated AST, elevated ALT, anemia, diarrhea, and weight gain (91). Merck’s MK-1084 showcased encouraging efficacy and a manageable safety profile in its Phase I trial presented at the 2024 ESMO Congress. In the monotherapy cohort, the drug achieved an ORR of 22% in patients with colorectal cancer, NSCLC, and cervical adenocarcinoma. The treatment was well-tolerated, with no dose-limiting toxicities reported (92). However, some drug development processes have faced challenges. JNJ-74699157 did not demonstrate significant clinical benefits during dose-escalation trials, and dose-limiting skeletal muscle toxicity was observed, leading to the halt of further development (93).

## 4 Exploration of resistance mechanisms to KRAS G12C inhibitors

Despite the initial success of KRAS G12C inhibitors, resistance has emerged as a significant challenge, driven by diverse mechanisms such as secondary KRAS mutations, MET amplification, and bypass pathway alterations (94). Acquired resistance, which develops during treatment through adaptive genetic and cellular changes, remains a key barrier to sustained efficacy. This section highlights acquired resistance mechanisms and their implications for improving therapeutic strategies.

### 4.1 Secondary KRAS mutations and amplifications

Secondary KRAS mutations, including alterations such as Y96D/S, G13D, and Q99L, represent a crucial mechanism of acquired resistance to KRAS G12C inhibitors (95). Among these, the KRASY96D mutation disrupts the switch-II pocket essential for inactive-state inhibitor binding, conferring cross-resistance to multiple KRASG12C inhibitors (94). Functional studies reveal differential susceptibility profiles among resistant clones—certain secondary mutations (e.g., Q99L) exhibit inhibitor-specific resistance patterns, suggesting potential utility in sequential therapeutic strategies (96). Emerging approaches demonstrate preclinical efficacy against these resistance mechanisms: the active-state inhibitor RM-018 maintains activity against KRAS G12C/Y96D, while BI-3406 (SOS1 inhibitor) combined with trametinib effectively targets Y96D/S-mediated resistance (97).

In addition to these secondary mutations, KRAS G12C amplification also contributes significantly to resistance by saturating KRAS G12C inhibitors through oncogene overload. Given the intrinsically high intracellular GTP: GDP ratio (~10:1), KRAS G12C amplification elevates total KRAS protein levels, disproportionately increasing GTP-bound active KRAS (94, 98). This increase in active KRAS offsets the inhibitory effects of KRAS G12C inhibitors, allowing the RAS-MAPK pathway to remain active.

### 4.2 RTK activation and compensatory signaling pathways

Activation of receptor tyrosine kinases (RTKs) is a major driver of resistance to KRAS G12C inhibitors. Genetic alterations, including amplification of EGFR, MET, and RET, as well as oncogenic fusions such as EML4-ALK and CCDC6-RET, reactivate the RAS-MAPK signaling pathway by restoring RTK pathway activity (94, 99). Notably, EGFR overexpression not only sustains the GTP-binding activity of KRAS G12C but also promotes resistance by activating wild-type RAS isoforms (HRAS/NRAS), leading to reactivation of the RAF-MEK-ERK cascade in a “two-pronged” resistance model (100). Preclinical studies have revealed that subclonal MET amplification in KRAS G12C inhibitor-resistant cells facilitates the conversion of RAS from its inactive GDP-bound state to its active GTP-bound state, thereby reactivating downstream signaling (101). In NSCLC cells, MET amplification not only induced RAS-mediated MEK-ERK activation but also activated the AKT pathway independently of RAS (101). Importantly, the MET inhibitor crizotinib effectively suppressed the RAS-MEK-ERK and AKT signaling pathways, restoring sensitivity to AMG 510 and achieving tumor regression in AMG 510-resistant xenograft models, underscoring the therapeutic potential of combining these agents (101). Additionally, genetic alterations such as RET amplification and oncogenic fusions, including EML4-ALK, CCDC6-RET, and TACC3-FGFR3, can directly reactivate the MAPK pathway, thereby driving drug resistance (94, 99). Targeting these alterations following the development of resistance to KRAS G12C inhibitors represents a promising avenue for future research and therapeutic development.

Resistance to KRASG12C inhibitors is not limited to mutations in the RAS-MAPK pathway but also involves significant reprogramming of compensatory signaling pathways. One key mechanism is the activation of the PI3K-AKT-mTOR pathway (95), driven by alterations such as gain-of-function PIK3CA mutations (e.g., H1047R) or PTEN loss, which lead to increased PI3K signaling and promote cell survival through the AKT-mTOR pathway. As previously mentioned, MET amplification drives resistance by activating both the PI3K-AKT and JAK-STAT3 pathways, providing alternative survival mechanisms. Preclinical studies have shown that combining MET inhibitors (e.g., crizotinib) with KRASG12C inhibitors can block these pathways and significantly delay the development of resistance (101).

RTK-mediated compensatory activation and intrinsic variations within compensatory signaling pathways create a dynamic network of resistance mechanisms. Addressing this challenge necessitates a combination of precise targeted therapies and continuous monitoring of tumor heterogeneity.

### 4.3 Co-mutations, histologic transformation, and EMT

Recent advances in understanding resistance mechanisms have uncovered additional factors influencing the efficacy of KRASG12C inhibitors, including co-mutations, histologic transformation, and epithelial-mesenchymal transition (EMT). Clinical studies have shown that concurrent mutations in KEAP1, SMARCA4, and CDKN2A are strongly associated with poor therapeutic responses to KRASG12C inhibitor monotherapy in patients with advanced NSCLC (102). Similarly, preclinical studies using KRAS G12C-mutant mouse models with STK11/LKB1 deletions have identified adenocarcinoma-to-squamous cell carcinoma transformation (AST) as a pathological feature linked to primary drug resistance (103). This histologic transition may contribute to the poor clinical outcomes observed in KRAS G12C/STK11 co-mutated patients and represents a potential therapeutic target for overcoming resistance to KRASG12C inhibitors. Furthermore, EMT is a well-recognized mechanism of adaptive drug resistance and subsequent cancer treatment failure. Studies have demonstrated that EMT may drive resistance to KRASG12C inhibitors through hyperactivation of the PI3K pathway (104). Consequently, strategies aimed at promoting mesenchymal-to-epithelial transition (MET) hold promise for overcoming this resistance. Additionally, immune factors may contribute to this process, and we will discuss how these immune factors are involved in the mechanisms of resistance to KRAS G12C inhibitors.

In conclusion, these findings underscore the multifactorial nature of drug resistance, offering valuable insights for developing predictive biomarkers and designing effective combination therapies.

## 5 The impact of KRAS mutations on the TME

**Figure 3:**
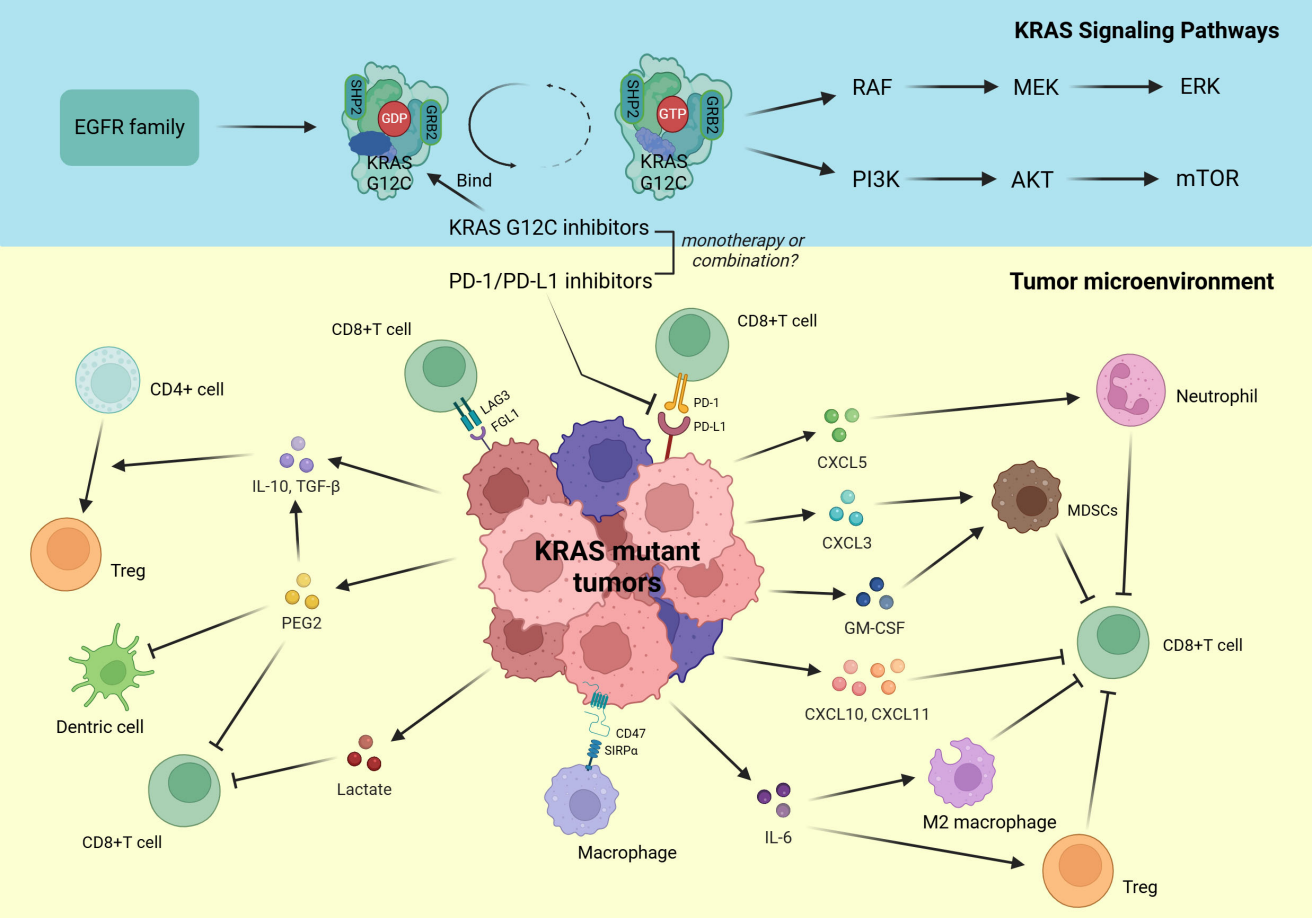
Upper Section, Persistently activated downstream signaling pathways of mutant KRAS. Lower Section, Tumor microenvironment influenced by mutant KRAS.

After examining the mechanisms of resistance to KRAS G12C inhibitors, it becomes essential to investigate how KRAS mutations influence the tumor immune microenvironment (TME). These mutations can trigger immune evasion, significantly affecting therapeutic efficacy (**Figure 3**). In the subsequent sections, we will delve into the effects of KRAS mutations on immunity and their implications for therapeutic strategies.

### 5.1 Modulation of immune checkpoints by oncogenic KRAS

Immune checkpoint molecules, such as PD-1/PD-L1 and CTLA-4, are critical mediators of tumor immune escape, enabling tumor cells to evade immune surveillance by binding to receptors on T cells and suppressing their activity. Emerging evidence indicates that KRAS mutations significantly influence the expression and function of immune checkpoint molecules by modulating specific signaling pathways (**Figure 3**). In a pivotal study, Chen et al. revealed significantly elevated PD-L1 expression levels in both tumor tissues and cell lines derived from KRAS-mutant lung adenocarcinoma patients, compared to their KRAS-wild-type, EGFR-mutated, and ALK-mutated counterparts (105). Mechanistically, the researchers identified that KRAS modulates PD-L1 expression specifically through the p-ERK signaling pathway, with minimal contribution from p-AKT (105). A seminal study has unveiled a profound and previously unrecognized mechanism involving Tristetraprolin (TTP), an AU-rich element (ARE)-binding protein, in the regulation of PD-L1 expression. TTP typically binds to AREs within the 3’ untranslated region (3’ UTR) of PD-L1 mRNA, thereby enhancing PD-L1 mRNA stability and negatively regulating PD-L1 expression (106). Notably, in the context of oncogenic KRAS mutations, the constitutively activated MEK signaling pathway induces TTP phosphorylation at specific serine residues, leading to its functional inactivation and subsequent dysregulation of PD-L1 expression (106). This intricate molecular crosstalk between KRAS-driven signaling and TTP-mediated post-transcriptional regulation not only provides novel insights into the mechanisms of immune evasion in NSCLC but also highlights potential therapeutic targets for modulating PD-L1 expression in KRAS-mutant tumors.

While the PD-1/PD-L1 axis remains a cornerstone of immune checkpoint regulation, accumulating evidence highlights the significant contribution of alternative inhibitory pathways in shaping the immunosuppressive TME of NSCLC. Emerging research particularly implicates lymphocyte activation gene-3 (LAG-3), cytotoxic T-lymphocyte-associated antigen-4 (CTLA-4), and T-cell immunoglobulin and mucin domain-containing protein-3 (TIM-3) as critical mediators of immune evasion (107). LAG-3 emerges as a central regulatory element - this activation-induced inhibitory receptor modulates immune homeostasis through coordinated suppression of CD4+ and CD8+ T-cell proliferation and effector functions (108). Mechanistic studies have established FGL1 as the principal functional ligand mediating LAG-3-dependent immunosuppression, with their interaction demonstrating potent inhibition of antigen-specific T-cell activation (**Figure 3**) (109). Recent studies reveal a marked inverse correlation between FGL1 expression levels and CD8+ T-cell infiltration density in KRAS-mutant LUAD (110). Mechanistically, oncogenic KRAS mutations activate the ERK1/2-SET1A signaling axis, leading to the sequential phosphorylation, stabilization, and nuclear localization of SET1A. In the nucleus, SET1A catalyzes YAP methylation, an epigenetic modification that drives its nuclear retention and enhances YAP-mediated transcriptional activation of FGL1 (110). This cascade establishes a pro-immunosuppressive tumor microenvironment, ultimately contributing to immune evasion in KRAS-mutant LUAD. Recent findings from studies in lung cancer cell lines and mouse models further elucidate this mechanism, demonstrating that the KRAS-ERK1/2-SET1A-YAP axis is central to FGL1 upregulation and the resulting immune escape phenotype (110). Notably, colorectal carcinomas with KRAS mutations display a distinct immunoregulatory profile, marked by the pronounced downregulation of immune checkpoints such as BTLA, CTLA-4, and TIGIT—a pattern in sharp contrast to that observed in pulmonary malignancies (111). This tissue-specific divergence underscores the pivotal role of the tumor’s originating microenvironment in shaping KRAS-driven immune checkpoint regulation.

### 5.2 Signaling pathways mediating immune evasion in KRAS-mutant tumors

#### 5.2.1 Ras-MAPK and PI3K/AKT pathways in immune evasion

In addition to directly regulating immune checkpoint expression, KRAS-mutated tumor cells have been shown to enhance the secretion of IL-10 and TGF-β1 through activation of the MEK-ERK-AP1 signaling pathway (112). These cytokines play pivotal roles in suppressing effector T cell activity and promoting the differentiation of regulatory T cells (Tregs) (**Figure 3**) (113). Silencing KRAS expression significantly reduces the secretion of these cytokines, accompanied by a marked decrease in Treg production (112). Interestingly, the Ras-MAPK pathway amplifies paracrine crosstalk between malignant cells and stromal components through CAF differentiation. Oncogenic RAS signaling facilitates CAF transformation via TGF-β-mediated mechanisms, with CAF-secreted CXCL5 subsequently activating the Ras-MAPK axis to upregulate PD-L1 expression and drive immune evasion (114, 115).

PI3K signaling maintains immunoregulatory potential in KRAS-driven tumors via distinct mechanisms. In KRAS G12D models, activation of the P70S6K-PI3K-AKT axis downregulates HMGA2 expression, correlating inversely with reduced CXCL10/CXCL11 chemokine production that impairs CD8+ T cell infiltration and antitumor immunity (116). Furthermore, this pathway facilitates myeloid-derived suppressor cells (MDSCs) recruitment through upregulated GM-CSF production, fostering an immunosuppressive niche via T-cell suppression (117). However, current evidence remains constrained to KRAS G12D preclinical models, creating critical knowledge gaps regarding PI3K pathway functions in KRAS G12C-driven malignancies that require systematic investigation.

#### 5.2.2 NF-κB, STAT3, and IFN pathways in immune evasion

KRAS-driven tumors employ multifaceted signaling networks that extend beyond canonical RAS-MAPK and PI3K-AKT pathways to include NF-κB, STAT, and interferon axes, each exerting critical immunoregulatory effects. KRAS mutations systematically orchestrate an immunosuppressive microenvironment through RAL-TBK1-IKKϵ-mediated NF-κB activation, driving IL-6 production that promotes accumulation of Tregs and protumorigenic macrophages while suppressing CD8+ T-cell effector functions (118, 119). In lung adenocarcinoma patient samples and KRAS-driven lung cancer mouse models, KRAS mutations engage PI3K/STAT3 axis activation to suppress miR-34a, resulting in transcriptional depression of the “don’t eat me” signal CD47. This weakens macrophage-mediated phagocytosis and promotes immune escape (**Figure 3**) (120). Therapeutic disruption of this axis through KRAS G12C inhibitors or CD47-neutralizing antibodies restores phagocytic competence, effectively reprogramming the immune contexture from immune-evasive to immune-responsive states (120).

Complementing these mechanisms, in KRAS-driven malignancies, suppression of the type I interferon (IFN) pathway emerges as a critical mechanism of tumor immune evasion, with pathway inactivation demonstrating strong correlation with concurrent activation of oncogenic signaling networks such as MYC (121). Current evidence indicates that type I IFN signaling deficiency manifests during early tumorigenesis in KRAS-mutant neoplasms and persists throughout disease progression (122). Mechanistic studies reveal that KRAS-MYC synergistic oncogenesis promotes MYC-mediated transcriptional repression of IFN-regulated genes (IRF5, IRF7, STAT1, STAT2) through formation of the MYC-MIZ1 inhibitory complex (121). Furthermore, MYC activation exerts dual immunosuppressive effects by downregulating JAK2 expression, thereby attenuating both type I and type II IFN signaling cascades and diminishing tumor cell responsiveness to interferon stimulation (123). Restoring IFN activity via STING agonists or IFN-β administration reverses immunosuppressive microenvironments by converting “cold” to “hot” tumor phenotypes, enhances B/NK cell infiltration, and synergizes with PD-1/PD-L1 blockade, demonstrating improved prognosis in preclinical models (122, 123).

### 5.3 KRAS mutations reprogram the tumor immune microenvironment

#### 5.3.1 Reshape cytokine and chemokine profiles to drive immune suppression

KRAS mutations profoundly reshape the TME by inducing the aberrant expression of cytokines and chemokines, leading to the suppression of effector immune cell functions (**Figure 3**). The CXCR2 signaling axis plays a pivotal role in this process, with the expression of multiple CXCR2 ligands (CXCL1/2/3/5/8) significantly elevated in KRAS mutant tumors, each contributing distinct immunosuppressive functions (124). CXCL8 (IL-8), a direct transcriptional target of KRAS, is strongly associated with tumor-associated inflammation and angiogenesis (125). Similarly, CXCL3 is upregulated due to KRAS-mediated inhibition of interferon regulatory factor 2 (IRF2), which exacerbates the recruitment of MDSCs in colorectal cancer (126). Additionally, SOX2-driven upregulation of CXCL5 in NSCLC promotes the accumulation of tumor-associated neutrophils (TANs), accelerating tumor progression (127). Together, these CXCR2 chemokines facilitate TANs and MDSCs recruitment into the TME, impairing CD8^+^ T cell function and contributing to immune suppression (124).

In parallel, KRAS mutations promote immune evasion through activation of the COX2/PGE2 signaling axis. PGE2 directly suppresses CD8^+^ T cell activity and dendritic cell (DC) antigen-presenting capacity, while also driving the expansion of Tregs by inducing IL-10 and TGF-β secretion (128, 129). This creates a positive feedback loop that sustains the immunosuppressive microenvironment. Moreover, KRAS mutations enhance the secretion of proinflammatory factors such as GM-CSF, which expand immunosuppressive Gr1^+^CD11b^+^ myeloid cells and further disrupt T cell-mediated immune surveillance (117). Preclinical studies emphasize that targeting the cytokine and chemokine networks, such as the CXCR2 or COX2 pathways, effectively reduces the immunosuppressive influence of MDSCs and Tregs in the TME, providing a promising strategy to counteract KRAS mutation-associated immune escape (124, 128).

#### 5.3.2 Recruitment of immunosuppressive Cells

KRAS mutations play a pivotal role in orchestrating an immunosuppressive tumor microenvironment by promoting the pathological recruitment of Tregs, MDSCs, and tumor-associated neutrophils via the upregulation of cytokine and chemokine expression (117, 124, 126, 127, 129). These immunosuppressive populations critically impair CD8^+^ T-cell infiltration and effector functions, thereby promoting immune evasion. In KRAS-mutant NSCLC, spatial multilabel analyses have demonstrated that the spatial interactions among immunosuppressive cells significantly contribute to resistance against ICIs. Notably, the spatial co-localization of CD68^+^ tumor-associated macrophages (TAMs) with FOXP3^+^ Tregs has been strongly associated with reduced ICIs efficacy, with non-nuclear FOXP3 expression further compromising therapeutic outcomes (130). These findings highlight the complex interplay of immune components within the KRAS-driven tumor microenvironment.

In addition, KRAS mutations directly target CD3^+^ T cells, a major subset within the T-cell population, thereby augmenting immune escape. The induction of CD3^+^ T-cell apoptosis by KRAS mutations not only leads to T-cell depletion and energy starvation but also undermines tumor immune surveillance, ultimately facilitating immune evasion by NSCLC cells (105). Notably, KRAS mutations upregulate PD-L1 expression and induce CD3^+^ T-cell apoptosis through the PD-1/PD-L1 axis, further reinforcing their central role in escaping immune destruction (105).

In conclusion, KRAS mutations profoundly reshape the TME through complex, multilayered interactions across diverse cell types. This remodeling enhances tumor immune evasion and poses significant challenges to the efficacy of immunotherapy.

### 5.4 KRAS co-mutations and tumor immune microenvironment regulation

STK11/LKB1 (KL), TP53 (KP), and CDKN2A/B inactivation combined with low NKX2-1 expression (KC) represent the major co-mutation patterns in KRAS-mutated NSCLC, each associated with distinct TME characteristics and differential responses to treatment (131). Among these, the KL subgroup exhibits an ‘immune-cold’ phenotype, characterized by reduced PD-L1 expression and diminished CD8^+^ T-cell infiltration (131, 132). This immunosuppressive environment is partly attributed to STING pathway silencing caused by LKB1 loss, FAK signaling-driven collagen deposition, and neutrophil accumulation, all of which suppress anti-tumor immunity (132–134). Additionally, elevated autophagic flux in KL tumors impairs antigen presentation by disrupting immune proteasome function (134). Mechanistic studies suggest that strategies such as using ULK1 inhibitors to restore antigen processing or inhibiting the IL-6 pathway can each significantly enhance the immunotherapeutic response in these tumors (134, 135). In contrast, the KP subgroup exhibits an ‘immune-hot’ phenotype, characterized by a high tumor mutational burden (TMB), elevated PD-L1 expression, and increased levels of inflammatory markers (131). TP53 mutations enhance the efficacy of ICIs by promoting neoantigen formation and activating adaptive immunity through the regulation of genes involved in DNA repair and cell cycle control (136). Furthermore, the suppression of mTORC1 signaling observed in the KC subgroup suggests that tumor-associated immune cells can undergo phenotypic and functional reprogramming within the tumor microenvironment (131, 137).

In addition to the previously discussed co-mutations, KRAS mutations in conjunction with KEAP1, Myc, and RNF43 alterations exert profound effects on the immune microenvironment. KEAP1 mutations stabilize EMSY, enabling immune escape by suppressing type I interferon signaling, even with increased mutational burden (138). Myc activation drives widespread tumor microenvironment remodeling, characterized by inflammation, angiogenesis, and immune suppression, with CCL9 and IL-23 signaling mediating effector immune cell exclusion (139). Similarly, RNF43 mutations reshape the tumor immune landscape through modulation of chemokine pathways such as CXCL5, further reinforcing immune evasion (140). Overall, KRAS co-mutations regulate the tumor immune microenvironment through distinct mechanisms, underscoring the necessity of optimizing personalized therapeutic strategies and validating their potential through preclinical studies.

### 5.5 Metabolic adaptation and immune effects in tumor microenvironment

KRAS mutations drive tumor survival, growth, and immune escape in colorectal, non-small cell lung, and pancreatic ductal adenocarcinomas by reprogramming cancer cell metabolism and reshaping metabolic interactions within the tumor microenvironment (141). KRAS mutations have been shown to drive high lactate production, resulting in its significant accumulation within the tumor microenvironment. This metabolic reprogramming directly triggers activation-induced cell death in activated CD8+ T cells, thereby weakening the anti-tumor immune response (**Figure 3**) (142). In pancreatic ductal adenocarcinoma (PDAC), KRAS further increases the metabolic demands of tumor cells by enhancing glycolytic pathways, which exacerbates local lactate accumulation and contributes to immune suppression (143). In summary, KRAS mutations reprogram metabolism to suppress immunity and promote tumor immune escape.

## 6 KRAS G12C inhibitors in combination with anti-PD-(L)1 therapy

### 6.1 Immunomodulatory effects of KRAS G12C inhibitors

Understanding the significant impact of KRAS mutations on the TME underscores the need to explore therapies targeting these mutations. KRAS G12C inhibitors, a promising treatment option, not only inhibit tumor cell proliferation but may also modulate immune cell function by altering the tumor microenvironment. Taking AMG 510 as an example, AMG 510 treatment significantly reduced tumor-infiltrating immunosuppressive cells, while enhancing the infiltration and activity of antigen-presenting cells and CD8+ T cells, thus activating adaptive anti-tumor immunity (31). Specifically, treatment resulted in a significant increase in total CD3+ T cells and CD8+ T cells accompanied by enhanced infiltration of macrophages and cross-presenting dendritic cells. AMG 510 also enhanced recruitment and activation of T cells and dendritic cells through upregulation of expression of chemokines, such as CXCL10 and CXCL11, resulting in a significant enhancement of immune surveillance within the tumor (31). Different KRAS G12C inhibitors exhibited mechanistically similar immunomodulatory effects. It was found that MRTX849 significantly enhanced anti-tumor immunity by up-regulating the expression of MHC class I proteins and suppressing immunosuppressive factors. In the KRAS G12C-mutated CT26 mouse model, MRTX849 reduced myeloid-derived suppressor cells in the tumor, while increasing the infiltration of M1-type macrophages, dendritic cells, and CD4+ and CD8+ T cells, demonstrating a significant tumor-shrinking effect (144). A similar phenomenon was observed in a mouse model of *in situ* lung cancer, using a multiplex imaging approach. similar phenomenon in a mouse model of *in situ* lung cancer (145). In addition to their primary mechanisms, KRAS G12C inhibitors have been shown to remodel the tumor microenvironment through additional processes. They boost interferon signaling, enhancing cytotoxic T-cell activity while reducing the infiltration of immunosuppressive myeloid cells (146). Furthermore, the inhibitors suppress COX2 and its downstream effector PGE2, restoring IFN-γ signaling (128). Together, these processes play a key role in reshaping the tumor microenvironment and enhancing anti-tumor immune responses.

However, prolonged use of KRAS G12C inhibitors may lead to the development of adaptive immune escape mechanisms in tumors, representing a potential immune-related aspect of resistance to KRAS G12C inhibitors. For instance, AMG510-resistant tumors exhibit an “immune cooling” phenomenon, characterized by a substantial reduction in CD8+ T cells, a decreased neoantigenic load, and an accumulation of mast cells (147). These changes are likely associated with KRAS-driven EMT and immunosuppression (148).

In summary, KRAS G12C inhibitors not only exert direct anti-tumor effects but also remodel the tumor microenvironment to enhance anti-tumor immunity. However, the emergence of adaptive immune escape mechanisms underscores the importance of exploring combination strategies, such as pairing KRAS G12C inhibitors with PD-1/PD-L1 inhibitors, to overcome these challenges.

### 6.2 The feasibility of combining KRAS G12C inhibitors with Anti-PD-(L)1 therapy

Evidence indicates that PD-1/PD-L1 inhibitors, as monotherapy or combined with chemotherapy, outperform chemotherapy alone in advanced KRAS-mutant NSCLC (149, 150). A retrospective analysis showed that pembrolizumab significantly improved OS and PFS compared to chemotherapy. In the KRAS G12C subgroup, pembrolizumab achieved a hazard ratio (HR) of 0.28 for OS and 0.27 for PFS, highlighting better outcomes relative to KRAS wild-type patients. ORR also favored pembrolizumab in both KRAS-mutant and G12C subgroups (149). These findings underscore the potential of PD-1/PD-L1 inhibitors in KRAS-mutant NSCLC, particularly in KRAS G12C variants. However, resistance to PD-1/PD-L1 inhibitors is an inevitable challenge, with mechanisms broadly classified into intrinsic and extrinsic factors. Intrinsic resistance involves tumor-specific alterations, such as intracellular mutations, low tumor mutational burden, and antigenic escape, which reduce tumor immunogenicity (151). Extrinsic resistance, on the other hand, arises from immune evasion within the tumor microenvironment, primarily mediated by immunosuppressive cells such as tumor-associated macrophages and myeloid-derived suppressor cells (151). As previously mentioned, the formation of an immunosuppressive tumor microenvironment and characteristics such as lactate accumulation in KRAS-mutant tumors may limit the efficacy of immune checkpoint blockade monotherapy (130, 142). Given the potential to reverse the immunosuppressive microenvironment, there is a strong rationale to explore combination strategies involving KRAS G12C inhibitors and PD-1/PD-L1 inhibitors, which may achieve synergistic effects. Notably, preclinical studies have already begun investigating the feasibility of such combinations.

In preclinical models, KRAS G12C inhibitors have shown significant synergistic anti-tumor effects when combined with anti-PD-1 therapy (31, 144). In immune-competent mice, AMG 510 not only significantly reduced tumor volume but also, when combined with an immune checkpoint inhibitor, produced durable curative outcomes. Notably, cured mice exhibited strong adaptive immune memory upon rechallenge with homozygous KRAS G12D tumor cells (31). Similarly, in the KRAS G12C-mutant CT26 syngeneic mouse model, although MRTX849 monotherapy significantly inhibited tumor growth and induced partial tumor regression, combination therapy with an anti-PD-1 antibody dramatically enhanced therapeutic efficacy (144). This approach induced a high proportion of durable complete responses (CRs) with no recurrence upon tumor cell re-inoculation, further validating the establishment of adaptive immunity (144).

However, the efficacy of this synergistic anti-tumor effect appears to be dependent on tumor immunogenicity. In highly immunogenic tumors with sufficient T-cell infiltration, combination therapy significantly prolonged progression-free survival and achieved durable tumor clearance through enhanced T-cell activation and increased pro-inflammatory factor expression (146). By contrast, in low-immunogenic tumors, while KRAS G12C inhibitors improved antigen presentation and T-cell activation by remodeling the TME, these changes failed to significantly enhance tumor sensitivity to immune checkpoint blockade (146).

Worth mentioning is the spatial enrichment of Tregs in immune-rejecting tumors, which may play a key role in limiting the efficacy of combination therapy. In these tumors, although KRAS G12C inhibitors promoted the activation and clustering of effector T cells (e.g., CD8+ T cells), frequent Treg aggregation and contact with effector T cells formed a localized immunosuppressive barrier (152). Experimental depletion of Tregs significantly improved the efficacy of KRAS G12C inhibitors combined with anti-PD-1 therapy, further enhancing the anti-tumor immune response (152).

In summary, the combination of KRAS G12C inhibitors and anti-PD-1 therapy demonstrated robust synergistic anti-tumor effects in preclinical studies, particularly in immunologically active tumors, by remodeling the tumor microenvironment to achieve durable tumor clearance and establish adaptive immune memory. However, optimizing immune strategies remains essential to enhance efficacy in low-immunogenic tumors. These findings provide a strong rationale and direction for clinical investigations of KRAS G12C inhibitors in combination with PD-1/PD-L1 inhibitors.

### 6.3 The clinical efficacy of combination therapy

Preclinical studies have demonstrated the feasibility of combining KRAS inhibitors with immunotherapy. Currently, multiple KRAS G12C inhibitors are being evaluated in clinical trials to explore the efficacy and safety of their combination with anti-PD-(L)1 therapies. The results of the relevant clinical trials are presented in **Table 3**.

**Table 3:** Clinical trials of KRAS G12C inhibitor combined with anti-PD-(L)1 therapy.

KRAS G12C inhibitors	Clinical tials	Anti-PD-(L)1 drugs	Control	Results
**AMG510 (sotorasib)**	CodeBreak100/101	Pembrolizumab/Atezolizumab	KRAS G12C inhibitor-naïve NSCLC	ORR 29% mDOR 17.9mo mPFS 15.7mo (95%CI, 9.8-17.8)
		Pembrolizumab	Lead in	ORR 37% (95% CI, 16-62) DCR 74% (95% CI, 49-91) mOS NE (95% CI, 10.1-NE)
		Pembrolizumab	Concurrent	ORR 32% (95% CI, 13-57) DCR 90% (95% CI, 67-99) mOS 14.1mo (95% CI, 6.2-17.8)
		Atezolizumab	Lead in	ORR 20% (95% CI, 3-56) DCR 90% (95% CI, 56-100) mOS 8.1mo (95% CI, 2.5-NE)
		Atezolizumab	Concurrent	ORR 20% (95% CI, 3-56) DCR 80% (95% CI, 44-98) mOS 11.5 (95% CI, 5.0-NE)
**MRTX849 (adagrasib)**	KRYSTAL-7	Pembrolizumab	PD-L1 TPS≥50%	ORR 63% (32/51) DCR 84% mDOR NE (95%CI, 12.6-NE) mPFS NE (95%CI, 8.2- NE)
**LY3537982 (Olomorasib)**	LOXO-RAS-20001	Pembrolizumab	KRAS G12C inhibitor-naïve NSCLC	ORR 78% DCR 100%
			NSCLC post-KRAS G12C inhibitor treatment	ORR 78% DCR 100%
	SUNRAY-01(NCT06119581)	Pembrolizumab		
**MK-1084**	NCT05067283	Pembrolizumab	PD-L1 TPS≥1%	ORR 71% (15/21)
**IBI351**	NCT05504278	Sintilimab		
**JDQ443**	KontRASt-01 (NCT04699188)	Tislelizumab		
**GDC-6036 (divarasib)**	Krascendo-170 lung(NCT05789082)	Pembrolizumab		
	NCT04449874	Atezolizumab		

Patients receiving AMG 510 monotherapy for 21 or 42 days before transitioning to a combination of AMG 510 and anti-PD-(L)1 drugs; Concurrent: Patients receiving a combination of AMG 510 and anti-PD-(L)1 drugs from the outset; PD-L1 TPS (Programmed Death-Ligand 1 Tumor Proportion Score): It represents the percentage of tumor cells showing positive staining for PD-L1.

#### 6.3.1 Based on AMG 510

In the CodeBreak 100/101 Phase Ib clinical trial, the efficacy of combining AMG 510 with Pembrolizumab or Atezolizumab was investigated in 58 patients with KRAS G12C-mutant NSCLC (21). Participants were divided into two cohorts: the Lead-in cohort, where patients received AMG 510 monotherapy for 21 or 42 days before transitioning to combination therapy, and the Concurrent cohort, where patients received the combination therapy from the outset. Across all evaluable patients, an ORR of 29% and a DCR of 83% were observed, with a median OS of 15.7 months. In the Lead-in cohort, the combination of AMG 510 with Pembrolizumab resulted in an ORR of 37%, a DCR of 74%, and a median OS that was not reached. Conversely, the AMG 510 plus Atezolizumab group displayed an ORR of 20%, a DCR of 90%, and a median OS of 8.1 months. In the Concurrent cohort, the AMG 510 with Pembrolizumab group exhibited an ORR of 32%, a DCR of 90%, and a median OS of 14.1 months. The group combining AMG 510 with Atezolizumab had an ORR of 20%, a DCR of 80%, and a median OS of 11.5 months. The incidence of grade 3-4 TRAEs in these groups was 53%, 30%, 89%, and 90% respectively, with increased liver enzymes being the most common TRAEs (21). Notably, the combination of AMG 510 with immunotherapy did not substantially enhance efficacy over AMG 510 monotherapy, and it was associated with a higher incidence of severe TRAEs. The Lead-in cohort demonstrated more enduring clinical efficacy and a better safety profile compared to the Concurrent cohort. These findings highlight concerns about the potential toxicity of combining AMG 510 with anti-PD-(L)1 therapies. Nonetheless, optimizing the sequence of administration might improve the outcomes of this regimen. AMG 510, as one of the foremost drugs under development, has shown promising results as a monotherapy, but further clinical evidence is necessary to ascertain the feasibility of its combination with anti-PD-(L)1 therapies.

#### 6.3.2 Based on MRTX849

Similarly, research on MRTX849 has evaluated its combination with anti-PD-1/PD-L1 therapy in previously untreated patients with KRAS G12C-mutant NSCLC. Recent results from the KRYSTAL-7 Phase II study by Mirati Therapeutics indicated that first-line treatment with MRTX849 in conjunction with Pembrolizumab presents promising antitumor efficacy and manageable safety. Among 51 patients with a PD-L1 tumor proportion score (TPS) of ≥50%, 32 achieved a confirmed response, resulting in an ORR of 63% and a DCR of 84%, with the median PFS not reached (22). The ORR for the combination therapy significantly exceeded that of Pembrolizumab monotherapy. Unlike AMG 510 combinations, the safety profile of MRTX849 and Pembrolizumab was consistent with each monotherapy, with only 4% of patients discontinuing due to TRAEs (22). Based on these findings, Mirati plans to launch a Phase III clinical trial to compare MRTX849 plus Pembrolizumab against Pembrolizumab alone as a first-line treatment in patients with a PD-L1 TPS ≥50% and KRAS G12C mutations. This trial merits ongoing observation.

#### 6.3.3 Based on LY3537982

At the 2023 AACR Annual Meeting, results from the LOXO-RAS-20001 study were shared, focusing on LY3537982 combined with ICIs in patients with advanced KRAS G12C-mutant solid tumors. Specifically, LY3537982 combined with Pembrolizumab was evaluated in NSCLC patients. Among KRAS G12C inhibitor-naive patients, the ORR was 78% and the DCR was 100%. For previously treated patients, the ORR was 25% and the DCR was 75%. Notably, lower doses (50/100 mg BID) of LY3537982 resulted in a 10% incidence of grade ≥3 TRAEs, compared to 66% in the higher dose group (150 mg BID), suggesting a more manageable safety profile at lower doses (79). Updated results from the low-dose cohort, including 44 patients, showed an ORR of 63% and a DCR of 93%, with median PFS not estimable. In patients with a PD-L1 TPS of ≥50%, the ORR reached 75%, while those with TPS <50% or unknown showed an ORR of 56%. TRAEs occurring in ≥10% of patients included diarrhea, elevated liver enzymes, fatigue, nausea, and itching (153). These findings underscore the significant potential of LY3537982 combined with Pembrolizumab, especially in KRAS G12C inhibitor-naive patients and those with high PD-L1 expression. The ongoing SUNRAY-01 trial continues to explore LY3537982 and Pembrolizumab across varied PD-L1 expression levels, with results highly anticipated.

#### 6.3.4 Based on MK-1084

Recent clinical data from a Phase I trial assessing MK-1084 in combination with Pembrolizumab in treatment-naïve patients with NSCLC expressing ≥1% PD-L1 revealed an ORR of 71% (92). This combination therapy demonstrated significantly improved efficacy and a manageable safety profile compared to MK-1084 monotherapy. TRAEs were reported in 79% of patients, with the most common being elevated ALT, elevated AST, and diarrhea. Notably, one instance of dose-limiting toxicity occurred in the group receiving 400 mg per day of MK-1084 alongside Pembrolizumab (92). While MK-1084 monotherapy yielded limited results, its combination with Pembrolizumab exhibited considerable efficacy. These unexpected findings spurred Merck to initiate a Phase III randomized, double-blind, multicenter trial to further evaluate the efficacy of MK-1084 in combination with Pembrolizumab versus Pembrolizumab plus placebo in previously untreated NSCLC patients with a KRAS G12C mutation and a PD-L1 TPS of ≥50%.

Moreover, outcomes for several combination immunotherapy regimens remain undisclosed. The KONTRAST-01 (NCT04699188) Phase Ib/II study includes cohorts assessing JDQ443 with tislelizumab. Concurrently, the Krascendo-170 Lung study (NCT05789082), a Phase I/II trial, aims to evaluate the efficacy and safety of GDC-6036 with Pembrolizumab, as well as GDC-6036 combined with Pembrolizumab, platinum-based chemotherapy, and pemetrexed (154). These strategies of combining KRAS G12C inhibitors with anti-PD-(L)1 drugs are expected to yield promising clinical outcomes, particularly important for enhancing the efficacy of KRAS G12C inhibitors and overcoming resistance.

### 6.4 Concerning safety issues

As with most combination therapies, the use of KRAS G12C inhibitors with anti-PD-(L)1 therapies results in increased toxicity. This has been evidenced in the CodeBreaK 100/101 trials, where the combination of AMG 510 with anti-PD-(L)1 therapies led to a high incidence of elevated liver enzymes, identified as the most common Grade 3-4 TRAEs. The occurrence of TRAEs was more frequent with the combination therapy than with either agent alone (21). The specific mechanism behind liver injury from this combination remains unclear. Notably, a case report by Parvin Begum et al. described a severe immune-related hepatitis in a KRAS G12C-mutant NSCLC patient previously treated with anti-PD-(L)1 therapies and subsequently with AMG 510. This suggests that AMG 510 might induce a pro-inflammatory state, contributing to such outcomes (155). Thus, mitigating TRAEs, particularly hepatotoxicity, is critically important. In the CodeBreaK 100/101 trials, corticosteroids effectively resolved most treatment-related adverse events. It was observed that lower doses of AMG 510 reduced the incidence of hepatotoxicity. Moreover, the Lead-in cohort experienced fewer Grade 3-4 TRAEs compared to the Concurrent cohort, indicating that the timing of AMG 510 initiation is pivotal in reducing hepatotoxicity (21). Several studies have indicated that patients receiving anti-PD-(L)1 therapies within three months prior to AMG 510 therapy are at an increased risk for Grade 3 or higher TRAEs, including hepatotoxicity, and are more likely to discontinue treatment due to adverse events (156, 157). A multicenter retrospective study found that patients in the Sequential group (who received anti-PD-(L)1 therapies as the last line before AMG 510) had a higher incidence of severe AMG 510-related TRAEs compared to the control group. Severe hepatotoxicity was notably more frequent in the Sequential group. Additionally, patients who had their last anti-PD-(L)1 drugs dose within 30 days before starting AMG 510 experienced a higher incidence of severe TRAEs (158). Therefore, while considering efficacy, the combination of AMG 510 and ICIs requires further research, with optimization of dosage, sequencing, and timing being crucial for controlling toxic reactions.

Attention is also focusing on the safety of other KRAS G12C inhibitor and anti-PD-(L)1 drug combinations. To date, combinations with MRTX849, MK-1084, and LY3537982 have displayed favorable safety profiles. In the KRYSTAL-7 Phase II trial, the combination of MRTX849 and Pembrolizumab showed a low incidence of treatment-related adverse events, with only 6% of patients discontinuing MRTX849 and 4% discontinuing both drugs due to adverse events. The incidence of elevated liver enzymes remained below 10% and was primarily low-grade (22). Similarly, the MK-1084 plus pembrolizumab regimen demonstrated a favorable safety profile. In Phase I, Grade 3-4 TRAEs occurred in 42% of patients, with elevated ALT and AST being the most common. No Grade 5 TRAEs were observed, and the combination was generally well-tolerated (92). For LY3537982 combined with Pembrolizumab, when LY3537982 was administered at 50 mg or 100 mg, the incidence of immune-related adverse reactions was relatively low. The predominant Grade 3 or higher TRAES was diarrhea, leading to discontinuation of any drug in 9% of patients and both drugs in 9% of patients (153). Interestingly, LY3537982 did not exhibit stronger hepatotoxicity compared to other KRAS G12C inhibitors; however, the small sample size necessitates larger studies for confirmation. Research on the combination of GDC-6036 and Pembrolizumab is ongoing, with preliminary findings yet to be published. Addressing the toxicity of combination therapies remains challenging, highlighting the need for further investigation into the mechanisms underlying these toxicities. Such research is vital to optimizing dosage and administration strategies to reduce adverse reactions.

## 7 Future perspective

The unique molecular structure and signaling role of KRAS have historically posed significant challenges in developing inhibitors targeting KRAS mutations, limiting clinical treatment options. However, precision targeted therapies have advanced rapidly, enriching our understanding of the biological characteristics and treatment strategies for NSCLC with KRAS mutations. KRAS G12C is the most prevalent KRAS mutation (10, 11) and, unlike other variants, it maintains GTPase activity akin to wild-type KRAS (28). This distinctive activation mechanism has enabled the development of direct inhibitors targeting KRAS G12C. Preclinical and clinical studies have demonstrated promising therapeutic efficacy for these inhibitors. Results from the CodeBreaK100 and KRYSTAL-1 clinical trials led to the FDA’s approval of AMG 510 and MRTX849 for NSCLC patients with KRAS G12C mutations who have experienced disease progression following platinum-based chemotherapy (40, 50). However, AMG 510 encountered challenges in Phase III trials, failing to significantly improve overall survival compared to docetaxel, thus not receiving final market approval (13). Several new KRAS G12C inhibitors, such as D-1553, JDQ443, and LY3537982, are under development, offering unique pharmacological properties and mechanisms of action. These drugs have shown antitumor activity in preclinical studies, potentially surpassing that of AMG 510 and MRTX849, thereby suggesting substantial potential for further development (57, 70, 78). Nonetheless, resistance to KRAS G12C inhibitors is unavoidable, and the mechanisms underlying this resistance are not yet fully understood, requiring further research. Exploring additional combination therapies is critical to overcoming issues of suboptimal efficacy and resistance following KRAS G12C inhibitor treatment.

Recently, RAS(ON) multi-selective inhibitors such as RMC-6236 and RMC-7977 have shown broad-spectrum anti-RAS activity in preclinical studies against various RAS gene mutations, including KRAS codon 12 mutations (159–161). These inhibitors offer potential for overcoming resistance through both monotherapy and combination approaches. The pan-KRAS inhibitor BI-2865 exhibits high-affinity binding to various KRAS mutations, showing potential in selectively inhibiting KRAS-dependent cancer cell signaling. Its related compound, BI-2493, has effectively suppressed tumor growth in KRAS-mutant models *in vivo* (162). A variety of inhibitors targeting KRAS pathway molecules have been developed, focusing on upstream factors like EGFR, SOS1, and SHP2, as well as downstream elements of the RAF-MEK-ERK/MAPK and PI3K-AKT-mTOR pathways. Additionally, targeting other molecules like CDK 4/6 further broadens the therapeutic approach. These inhibitors have demonstrated efficacy on their own and are increasingly being tested in combination with KRAS G12C inhibitors. This approach aims to improve treatment outcomes by potentially overcoming resistance mechanisms and enhancing antitumor efficacy (163). Notably, anti-EGFR antibodies, SHP2 inhibitors, and FAK inhibitors particularly have shown significant effects in clinical trials when used alongside KRAS G12C inhibitors (62, 66, 164). For example, the combination of D-1553 with the FAK inhibitor ifebemtinib demonstrated an ORR of 87.5% and a DCR of 93.8%, indicating considerable potential (62). Combining KRAS G12C inhibitors with pathway-targeted inhibitors remains a promising avenue for future research.

Current guidelines recommend chemotherapy combined with immunotherapy as the first-line treatment for advanced NSCLC without driver mutations. The introduction of immunotherapy has benefited patients with KRAS mutations by modifying T cell function and the immune microenvironment. These immune effects, post-KRAS inhibition, have been extensively studied (149, 165, 166). Combining KRAS G12C inhibitors with immunotherapy appears feasible. Clinical trials have explored anti-PD-(L)1 therapy combined with drugs like AMG 510, MRTX849, MK-1024, and LY3537982 (21, 22, 153). Notably, MRTX849 with pembrolizumab achieved an ORR of 63% and a DCR of 84%, significantly enhancing the efficacy compared to MRTX849 alone (22). A first-line treatment with LY3537982 combined with pembrolizumab resulted in an ORR of 78% and a DCR of 100%, indicating remarkable efficacy (79). However, this impressive efficacy comes with unavoidable toxicities, particularly hepatotoxicity (21, 22). Addressing the toxicity of these combinations without compromising efficacy remains a critical challenge. Current research suggests that optimizing administration regimens and dosing of combination therapies might partially reduce these toxicities (21). Selecting suitable drug combinations based on the pharmacological properties and mechanisms of KRAS G12C inhibitors and anti-PD-(L)1 drugs, validated through preclinical studies, can help maximize therapeutic benefits while minimizing adverse effects. Overall, the combination of KRAS G12C inhibitors with immunotherapy continues to hold substantial promise.

## 8 Conclusion

The KRAS G12C mutation is a significant driver mutation in non-small cell lung cancer, with its unique biological properties and clinical relevance positioning it as a focal point for targeted therapies. Recent advancements in targeted treatments, such as AMG 510 and MRTX849, have shown promising efficacy and received FDA approval, marking significant progress for patients with this mutation. However, despite the effectiveness of these inhibitors in monotherapy, the emergence of resistance mechanisms—such as secondary mutations and bypass signaling activation—poses a considerable challenge in clinical settings.

Additionally, the immunomodulatory role of KRAS mutations within the tumor microenvironment is becoming increasingly recognized. Studies indicate that KRAS mutations can facilitate immune escape and influence patient responses to immune checkpoint inhibitors by modulating the expression of immune checkpoint molecules, activating downstream signaling pathways, and altering the infiltration of specific immune cells. This understanding supports the theoretical basis for combining KRAS G12C inhibitors with anti-PD-(L)1 therapy. Recent clinical data demonstrate that this combination can yield synergistic effects in certain patients, enhancing the objective response rate and prolonging progression-free survival. Nonetheless, further exploration is needed regarding the safety of this combination therapy, optimal dosage regimens, and effective biomarker screening.

Future research should focus on the immune effects of KRAS G12C mutations, mechanisms of drug resistance, and strategies to optimize combination therapies. Moreover, tailored therapeutic approaches that consider individual patient characteristics—such as genomic factors and the immune microenvironment—are anticipated to enhance therapeutic efficacy. As more clinical trial data are generated and novel treatment methods are developed, the prospects for patients with KRAS G12C mutated NSCLC look increasingly promising.
